# Improving the visibility and description of theory in qualitative research: The QUANTUM typology

**DOI:** 10.1016/j.ssmqr.2021.100030

**Published:** 2022-12

**Authors:** Caroline Bradbury-Jones, Oliver Rudolf Herber, Rosemarie Miller, Julie Taylor

**Affiliations:** a School of Nursing, Institute of Clinical Sciences, University of Birmingham, B15 2TT, UK; b Witten/Herdecke University, School of Nursing Science, Alfred-Herrhausen-Str. 50, 58455 Witten, Germany; c Heinrich Heine University Dusseldorf, Institute of General Practice, Moorenstr. 5, 40225 Dusseldorf, Germany; d Birmingham Women's and Children's NHS Foundation Trust, UK

**Keywords:** ADAPT-ITT, Description, Theoretical framework, Theory, Typology, Qualitative, QUANTUM, Research, Reporting

## Abstract

The relationship between theory and qualitative research has been much debated. In 2014, based on an analysis of qualitative studies, we published a five-point typology on the levels of visibility expounded in such studies. The typology captured a range of theoretical visibility – from seemingly absent to consistently applied. In 2020, we undertook a project to critique and revise the typology, guided by the ADAPT-ITT framework. ADAPT-ITT was developed originally to inform the adaptation of evidence-based interventions to new geographic regions, cultural contexts or populations related to HIV. It has subsequently evolved as a helpful framework in a number of health and social fields. The ADAPT-ITT framework provides a systematic, stepwise process that allows existing interventions to be adapted, rather than creating new interventions unnecessarily. The use of ADAPT-ITT to guide the adaptation of a methodological framework (as opposed to a health intervention) is novel and we used it flexibly, as reported in this article. Core to this process was the engagement of 14 international qualitative research experts, drawn mainly from health and social science disciplines. The outcome was a revised typology, presented in this article. We offer this as a reflexive aide for the conduct and reporting of qualitative research.

## 1 Introduction

The relationship between qualitative research and theory is both complex and contentious and numerous scholars have alluded to lack of consensus and poor understandings that reflect this troubled pairing (Sandelowski 1993; Anfara & Mertz 2006; Tavallaei & Abu Talib 2010; Wu & Volker 2009). The problem seems to be that the role of theory in qualitative research is variable and can be used in different ways (Biesta, Allan & Edwards 2011; Brown, Bearman, Kirby, Molloy, Colville & Nestle 2019). Creswell and Poth (2017) observe that the landscape of qualitative research has changed and the qualitative enterprise has become more fragmented. They argue that qualitative researchers are far more aware of the designs they are using than they were in the 1990s and that they face a baffling number of choices. Additionally, varying definitions of theory exist and researchers tend to use the same words to mean different things (Wu & Volker 2009). The problem is that ‘terms slip and slide, fall over one another’ (Denzin 2017, p. 8). In qualitative research a variety of terms have come to the fore, which can be extremely confusing, especially for neophyte researchers. Contention, lack of consensus and fragmentation risk qualitative research being regarded as an incoherent endeavour which exposes it to charges of lack of theoretical robustness and maturity. In 2006, Anfara and Mertz highlighted the criticisms levied against qualitative research for its tendency to lack theory in its development or conduct. Their review of theoretical frameworks in qualitative research found little uniformity regarding the role of theory and it was often non-existent. This is echoed by the more recent work of Delaisse and Huot (2020), who undertook a literature review focusing on the use of theory in occupational science, including 41 studies on global migration. Their analysis revealed inconsistencies in the application of theory with some articles not explicitly using any theoretical concepts. Similarly, O'Leary and colleagues (2020) state that although the use of theory in qualitative enquiry adds depth to research and increases the transferability of findings, it is often *ad hoc*, superficial and poorly reported. To support meaningful theory application, they recommend consistent consideration of theory in reporting and quality appraisal tools. Gülpinar, Keleş, & Yalim (2021) provide an example of a theory-driven qualitative study with community pharmacists. Their study, employing the theory of planned behaviour, provided an in-depth understanding of factors that determine pharmacists' refusal to offer services contrary to their personal beliefs in an Islamic country.

Sperka (2019), a doctoral candidate, details how she grappled with the role of theory in qualitative research and offered valuable strategies to novice researchers for negotiating theoretical tensions. Wu and Volker (2009) suggest that qualitative researchers do not articulate consistently how theory has been applied. This really is the crux of the matter. Sandelowski (1993) asserted several years ago, that there is a need for qualitative researchers to ‘unmask theory: to recognize it in its many guises and disguises’ (p. 217). Echoing this, as we have argued previously in Bradbury-Jones et al. (2014), the problem is not so much lack of theory *per se*, but rather lack of identification and *articulation* of the theory.

This article is intended as a contribution to the field of qualitative research, particularly in health and social sciences, that assists in the unmasking of theory. As Biesta et al. (2011) observe, theory is difficult to define and therefore, before describing our project it is important to be clear about our own terminology. As Collins and Stockton (2018) point out:

‘theory, theoretical frameworks, theory of method, and conceptual frameworks are terms that have blurred lines within qualitative methods literature and either suffer or benefit from widespread nuanced differences’ (p.2).

These authors put forward a helpful explanation of such terms that aligns with our own use in this article: A theory is a big idea that organizes many other ideas. Theory of method (methodology) provides guidance in relation to the methods, whereas a conceptual framework acts as a map of how all the literature drawn upon within a particular study, works together. A theoretical framework is the use of a theory (or theories) in a study that provides ‘a clearly articulated signpost or lens for how the study will process new knowledge’ (Collins & Stockton 2018, p. 2). While it is important not to become hamstrung by grappling with different terminologies, it is necessary to understand that different terms exist and moreover, that they are frequently used interchangeably.

## 2 Background

In 2014, based on a robust analysis of qualitative studies, we published a typology on the levels of visibility expounded in qualitative studies in the context of health and social sciences (Bradbury-Jones et al. 2014). The typology consists of five levels (Table 1). These capture what we called ‘the degree of visibility of theory’ - that is - the levels in which theoretical frameworks underpinning the research are made explicit within the publications. The typology captured a range of use of theory from Level 1 (where theory is seemingly absent) through to Level 5 (where theory is consistently applied, throughout the entire research process).

**Table 1:** Levels of Theoretical Visibility Typology (Bradbury-Jones et al. 2014).

Level of theoretical visibility	Descriptor
**Level 1:** Seemingly absent	Theory is not mentioned at all.
**Level 2:** Implied	Theory may be mentioned or discussed in some detail (mainly in the background and/or introduction sections) and reference might be made to theorists in the field, but no explicit statement is made about the influence of these on the study.
**Level 3:** Partially applied	Researchers explicitly locate their study within a particular theory but then seem to abandon efforts to link, apply or interpret their findings in that context. Theory is used only partially throughout the research process in relation to the research aims, interview questions or data analysis.
**Level 4:** Retrospectively applied	Theory is considered at the end of a study as a means of making sense of research findings. Theory may be introduced as an afterthought.
**Level 5:** Consistently applied	Theory is consistently applied throughout the entire research process. Theory guides and directs the various phases of the research process and can be tracked throughout a published article.

In 2020, we undertook a project to critique and revise the typology. It was part of a larger, Wellcome Trust funded study in the UK, known as the Qualitative Network for Theory Use and Methodology (QUANTUM) https://wellcome.org/grant-funding/people-and-projects/grants-awarded/establishing-qualitative-research-network-theory Core to the project was the engagement of a panel of 14 international qualitative research experts drawn from health and social science disciplines. In this article we present the principal output (the revised typology – renamed as the QUANTUM typology) along with a detailed description of the processes undertaken to achieve its revision.

## 3 Data and methods

We structured the revision process with reference to the ADAPT-ITT framework described by Wingood and DiClemente (2008). This mnemonic encompasses eight aspects: **A**ssessment, **D**ecisions, **A**dministration, **P**roduction, **T**opical Experts**, I**ntegration, **T**esting, **T**raining. It was developed originally to inform the adaptation of evidence-based interventions to new geographic regions, cultural contexts or populations related to HIV (Latham et al., 2010, 2012; Wingood, Simpson-Robinson, Braxton, & Raiford, 2011). ADAPT-ITT has subsequently evolved as a helpful framework in other fields such as sexual violence prevention (Munro-Kramer et al., 2020) and telehealth for intimate partner violence (Jacks et al., 2020). A strength of the ADAPT-ITT framework is that it provides a systematic, stepwise process that encompasses the key elements of an adaptation. Additionally, it allows existing interventions to be adapted, rather than creating new interventions unnecessarily. The use of ADAPT-ITT to guide the adaptation of a methodological framework is novel and we used it flexibly, as reported in this article. Table 2 shows the original understandings associated with ADAPT-ITT as described by Wingood and DiClemente (2008).

**Table 2:** The (original) ADAPT-ITT framework.

Phase	Key questions	Methodology
**A**ssessment	Who is the new target population?	Conduct focus groups/needs assessment with the new target population
**D**ecisions	What intervention is going to be selected and is it going to be adopted or adapted?	Decide on whether to adopt or adapt
**A**dministration	What in the original intervention needs to be adapted and how should it be adapted?	Administer a ‘theatre test’ with members of the new population
**P**roduction	How do you produce the first draft documenting the adaptations to the intervention?	Produce a first draft of the adapted intervention Maintain fidelity to the core elements and underlying theoretical framework to the original intervention Develop an adaption plan Develop quality assurance and process measures
**T**opical Experts	Who can help to adapt the intervention?	Identify and actively involve topical experts
**I**ntegration	What is going to be included in the adapted intervention that is to be piloted?	Integrate content from topical experts and create second draft of adapted intervention Assess content and readability of new intervention and develop third draft
**T**raining	Who needs to be trained?	Train staff to implement new intervention
**T**esting	Was the adaptation successful and did it enhance outcomes?	Undertake pilot studies

In Table 3 we present the ADAPT-ITT framework applied to our study. The left-hand column shows the original eight phases and these are juxtaposed with our interpretations in the right-hand column. The article is weighted towards detailing the work with the topic experts because this is where the justification for the adaptation was pivotal.

**Table 3:** The modified ADAPT-ITT Framework applied to our project.

Phase	Methodology
**A**ssessment	Sought feedback from qualitative researchers and post-graduate students about the clarity and utility of the typology
**D**ecisions	Based on critical feedback, decision taken to adapt the typology
**A**dministration	Team meeting to discuss the potential modifications required to the typology and the methodology to be used to achieve it
**P**roduction	Produced copies of the original typology containing preliminary suggestions for modification based on initial feedback
**T**opical Experts	Identified and actively involved qualitative research experts to critically review the original typology containing preliminary suggestions for modification
**I**ntegration	Integrated content from the qualitative research experts to create a revised draft of the typology
**T**raining	The revised typology integrated into teaching
**T**esting	Ongoing feedback from qualitative community and students

**Table 4:** Points of consideration sent to the expert panel.

The purpose of the Typology is to provide a framework through which the relationship between theory and qualitative research can be understood. To what extent does the Typology achieve this purpose? Please give reasons. In the article the five levels of visibility indicate the levels at which theories are made explicit within a sample of academic publications. What are your thoughts regarding the five levels? Can you think of other levels of visibility? How might the five levels be improved? What resources/tools, if any, do you currently use to review the use of theory when peer reviewing articles or supporting students and researchers in their report writing? How, for example, do you gauge the clarity with which theory is articulated and reported? How might the Typology be used by researchers in the writing process? For example, are the five levels transferable from judgements about publications to the work of student researchers? How might the Typology be used by external experts involved in quality assurance at specific stages of the research process? What are the limitations and shortfalls of the Typology?

### 3.1 Assessment

The original typology was intended as a resource for postgraduate students and early career researchers venturing into the field of qualitative research as novices. We have used the typology extensively in our own qualitative methods classes in health and social care higher education in the UK. As part of the ADAPT-ITT process, we reflected on the feedback from students on a taught qualitative methods course at University of Birmingham, on which CB-J was an instructor. Through formal and informal feedback in classroom discussions and tutorials with students, they have asked us frequently about certain aspects of the typology. Principally, these have been normative questions: Is ‘retrospectively applied’ bad? Is ‘consistently applied’ always the best? Should the levels of the typology be interpreted as hierarchical? Is there a place for a-theoretical qualitative research? Such questions stimulated our awareness of some of the ambiguities of the original typology that strengthened our rationale to undertake a revision.

### 3.2 Decisions

In 2019, we took the decision that after five years of publication, the typology was due for an assessment of its utility and currency.

### 3.3 Administration

We held a team meeting to discuss the potential modifications required to the typology and to identify an appropriate process and methodology through which the revisions could be achieved (i.e. ADAPT-ITT).

### 3.4 Production

We produced copies of the original typology that were then used in the following parts of the ADAPT-ITT process.

### 3.5 Topical experts

Through our extensive national and international networks, we invited 14 qualitative research experts to take part virtually in this pivotal phase (four from USA; one from Austria; and nine from the UK). They formed a panel drawn from the fields of applied health research, medical sociology, physical education and sport, nursing, social policy, sociology, applied psychology and criminology (see acknowledgement section of this article for details of the panel). We had initially planned to hold a two-day, face-to-face workshop, but due to the challenges posed by the COVID-19 pandemic, we used the digital technologies of email and Zoom. This comprised two stages:

1. Each member of the expert panel was sent an email containing the original, published article. The email contained a link to a recorded presentation by the lead author that provided context to the project and a request for feedback on the points of consideration presented in Table 4. This took place in April 2020. All members of the panel returned feedback via email.
2. In May 2020 we held two, 90-min interactive sessions via Zoom, with approximately half of the expert panel attending each session. The reason we divided them in this way was to facilitate interaction in a smaller group, with the opportunity for more contributions. The purpose of the sessions was to generate further discussion about potential adaptations to the typology, provide a forum for experts to meet one another and to discuss and debate some of the issues raised. Participants gave permission to record the online workshops using the local recording feature offered by Zoom https://support.zoom.us/hc/en-us/articles/201362473-Local-recording

Through these two stages we amassed considerable, invaluable information. Email feedback on the typology generated 23 pages (A4 size) of comments, annotated articles, and suggestions from some of the experts for useful resource material. Additionally, we had generated 180-min of Zoom recording from the online workshops (along with ‘chat’ boxes in text format). Unexpectedly, the forced use of digital technologies had generated far more information than we had anticipated. To allow us to do it justice and turn ‘information’ into ‘data’, we applied for ethical approval from the Science, Technology, Engineering and Mathematics Ethical Review Committee at the University of Birmingham, which was granted.

### 3.6 Integration

Data from the different stages (as already described) were thematically analysed using an inductive approach (Braun & Clark, 2006, 2019). As a first step, CB-J led the analysis and the identified themes were then discussed and agreed with the other three team members. This cross-checking aligns with what Campbell, Quincy, Osserman and Pedersen (2013) describe as intercoder agreement; where two or more coders reconcile through discussion any coding discrepancies. We considered it an important step in enhancing the rigour of the analysis. This analytical process led to five themes: 1. Relationship between theory and qualitative research; 2. A matter of values and assumptions; 3. Balancing inductive and deductive approaches; 4. A place for different types and emerging theories; 5. Collapsing and revising the levels. The reporting of themes is supported by illustrative examples from the data that show the spread across all experts. Each expert was assigned an individual code to protect their anonymity. Most quotes are drawn from the email feedback, supported by data from the online workshops as indicated.

#### 3.6.1 Theme 1: relationship between theory and qualitative research

Six experts pointed to the limitations of the typology as regards the relationship between theory and research:

I think the Typology indicates the extent to which theory has been considered and reported, and where this has been done, but I am not sure it helps us to understand in any detail the relationship between theory and research, which I think can be iterative and 2-way, and involves a process of understanding and application (expert 8)

Here, the expert viewpoint was that the original typology failed to take account of the iterative and two-way relationship between theory and qualitative research. . This was supported by other views about judgements of quality:

I think that the Typology doesn't really explore the relationship between theory and qualitative research *per se* … Rather it reports a continuum of perceived poor to best practice in articulating the use of theory within a primary qualitative study […] The Typology provides an opportunity to place primary qualitative studies onto that continuum dependent on how the authors have articulated the use of theory within their study and how this articulation has been judged as transparent or visible by an assessor (expert 9)

In sum (and supported yet further by the following quotes), we gained important insights into the perceived over-simplification of the typology in terms of capturing the complex relationship between theory and qualitative research:

I do not think the Typology offers a way to understand the relationship between theory and qualitative research. Essentially, the Typology suggests that papers consider theory somewhere between not at all or consistently. The nature of the relationship, or connection, between theory and qualitative research is not really explored at all (expert 10)

The Typology shows the levels of visibility rather than the relationship between theory and qualitative research which is more complex. Perhaps rather than relationship, it shows the ways in which theory is applied to written qualitative research, because as the authors point out, theory may be present, just not articulated (expert 13)

One of the workshop participants pointed out that the theory is not the core issue, it is the discovery of qualitative research that is important:

I thought that this [typology] was very useful and I could see myself using it with PhD students, and early career researchers, and it made me think in different ways … But actually, there is something really important about qualitative research and that is that the stuff gets in there is a whole new phenomenon, you know, there is no theory – you are discovering something totally new and that is one of the strengths of qualitative research (expert 5, online workshop).

#### 3.6.2 Theme 2: a matter of values and assumptions

To some extent, all our experts commented on the normative aspects of the typology, pointing out its inherent problems in stimulating value judgements. The language used in the following quotes, such as ‘best, good, desired, optimal, problematic, negative’ etc., illustrate concerns about the typology and as the last quote states, a call for it to become less value-laden:

The levels suggest that 5 is best (because it's the highest level). I'm not sure that's always the case. Does a ‘good’ qualitative study always need to have an identified theory upfront guiding the study? (expert 4)

The Typology feels more like critical appraisal than reporting. I may be misunderstanding, but it seems that level 5 is the level desired and therefore would result in a paper receiving a higher score. (expert 3)

One expert pointed out the typology's harsh stance towards post-hoc theory, drawing on their own experience of theory often becoming apparent some way into the research process:

I am concerned about them [the levels] becoming hierarchical. I would hate for this to be yet another high bar for qualitative researchers to aspire to when conducting their work […] Particularly the somewhat harsh stance about post-hoc theory involvement – I have seen many people not know what theory to use until they get their data analysed and then one becomes apparent. I don't think it deserves the negative portrayal here … Try to find a way to be less value-laden (expert 7)

Echoing these points about value judgements, most experts expanded their feedback to put forward suggestions for how these could be mitigated, as in the following examples:

There is an underlying assumption in the article that Level 5 is optimal and desired in all qualitative research and that the lower levels are problematic. While I feel that theory is very important in qualitative research, I would challenge the authors to consider circumstances when Levels 1–4 are acceptable in qualitative research – perhaps for certain types of qualitative studies, or objectives. Perhaps aligning the objectives of the qualitative study to the Levels would help with this (expert 13)

I am also slightly worried about the ‘seemingly a-theoretical’ studies – is there a space for the “descriptive qualitative” study where the main aim is to elicit and summarize the participants' perspectives on a particular topic/experience/service? (expert 2)

An important point was that value judgements made about the different levels, take no account of whether use of theory is robust.

It doesn't make sense to me that you can potentially be in level 5 as the use of theory is well articulated but there is no assessment of whether the use of theory was methodologically robust (expert 9)

The issue of robustness is reinforced by another expert who describes the issue of trying to find a theory to fit the data and refers to the example of digital technologies such as social media, which use methods of interacting that are different from face to face contexts.

When you start with a theory, and this is particularly the case with digital technologies when new things start emerging, you try to squeeze the data into a theory because you feel like it has to fit. And then you're not explaining what you found at all, you're just doing it to fit the theory (expert 12)

#### 3.6.3 Theme 3: balancing inductive and deductive approaches

A prominent theme within all the data was how to balance inductive and deductive approaches in qualitative research. One expert expressed concern and puzzlement about where ‘truly inductive’ approaches fit within the typology:

[The Typology] makes most sense to me when thinking about specific theories and I struggle a bit with using the Typology for studies that truly take an inductive approach (start with a problem or puzzling observation/phenomenon and work to develop an understanding of it). They likely would not achieve Level 5, as I understand it. Actually, I'm not sure where such studies might fit … (expert 3)

Extending their point, this expert made a suggestion for revision:

Perhaps the Typology needs a version for qualitative inquiry that is truly inductive (perhaps with just a general orientation or framing of the phenomenon of interest) vs. qualitative inquiry that is guided by a specific theory (expert 3)

We received similar feedback from expert 6 pointing out the limitations of advocating for solely inductive approaches. Reference to what they term ‘data that fall outside of the initial theoretical premises’:

I'd have liked to see more acknowledgements of the ‘limitations’ of a theory driven approach to qualitative research: a purely deductive approach can be problematic as it ignores the data that fall outside of the initial theoretical premises (expert 6)

#### 3.6.4 Theme 4: a place for different types and emerging theories

Within themes 1–3 we captured the disquiet of all the experts (to some degree) about the restrictive nature of the original typology and as already reported the complexity of balancing relationships not only between theory and research, but also inductive and deductive approaches related to the qualitative endeavour. We discerned another related and prominent theme that warranted a separate space in the reporting. As a result of comments from almost all the experts, we were challenged to consider the place of different types of theory and those that emerge from the research.

Or another useful way of looking at it is the distinction between grand theories (overarching conceptual theories, unified theories about the social world), mid-range theories (I think CMS would be categorised here - a patterned set of hypotheses or assumptions about how things in a focused area are connected) and programme theories (small theory about how a policy or programme is intended to work) (expert 4)

I wanted clearer signposting that the Typology referred to substantive theory rather than paradigmatic theory (expert 6)

Our analysis led us to question whether four dimensions of theory might be more appropriate than levels for evaluating research. How, for example, has the researcher explored and examined prior research in the field relevant to the research question? (a process which the expert refers to as researching the ‘state of the art’). What is the philosophical logic, or which particular theories are being used? What discipline is the researcher coming from? (e.g. psychoanalysis, behaviourism, cognitive psychology), and which methodology is being used (e.g. ethnography, grounded theory etc.)

It would be very fine to differentiate the five levels on all four of them [dimensions]. On all four dimensions you can reach a high level of theory or a low level of theory (expert 14)

The place of emerging theories was also questioned as per these three expert viewpoints:

Suppose a study begins with a general orientation (perhaps identifying a grand theory) and then either identifies a more specific theory that fits or develops a new theory or framework from the data. Where would this fit in the Typology? (expert 3)

There may be really good reasons why theory wasn't used all the way through. It might be that the most useful aspect of using theory was the theory that was specifically applied to understand the findings. And to me that's the crux of it. Whether using theory helped you to understand your data better and also, just as important to me was whether your data helped us to understand the theory better. You know, because I feel that we shouldn't see theories as kind of undeniable tablets of stone. They ought to be modified and exemplified and improved and refined or disproven all the time (expert 4 online workshop)

The strength of the qualitative paradigm is that it encourages ‘thinking outside the box’ – in this case that might be outside pre-existing substantive theory from the discipline in question or empirically driven research that generates novel theory (expert 6).

#### 3.6.5 Theme 5: collapsing and revising the levels

We had asked the experts to suggest modifications to the typology and the themes already presented capture many of their suggestions. This final theme focuses on their suggestions to collapse and revise the five levels of the typology to mitigate its limitations:

I did consider whether 1 and 2 could also be collapsed … but think it's important to separate ‘no mention’ of theory from ‘implied’ mention of theory … I found myself wanting to collapse the 2 categories ‘Partially applied’ and ‘retrospectively applied’ into one category of ‘Partially applied’ (expert 6)

I wondered about level 4 and whether it's different from level 3. I'm not sure it's necessary that it’s an afterthought (bit judgemental in tone) rather than that it’s applied to analysis and interpretation but not to earlier stages. That might not be an afterthought – it might have been the plan (rightly or wrongly) all along (expert 4)

One question I have is about conceptual overlap. For example, is level 4 just a sub-set of level 3? (expert 10)

Several experts pointed out the specific problems associated with level 4, particularly given that it is so hidden and difficult to assess objectively:

The retrospective application of theory (level 4) is one that I think we all intuitively know about/suspect in articles. I appreciate it being present in the Typology, as it unveils a strategy used in studies that appear to lack theoretical rigor. Its presence in the typology may then give pause to researchers who use theory in this way (e.g. as post-hoc justifications). However, the practical application of level 4 seems problematic. It is much less practical than the other levels, because, as the authors point out? you never really know that this was done in the way suggested … My concern then is how useable is this level if it can't be assessed (expert 13)

While one expert questioned the practical application of retrospectively applied theory, another put forward the argument that it may risk encouraging researchers to be deceitful in their reporting – clearly a practice that we had not intended to suggest:

For many researchers, the lack of theory at the beginning is not so much a weakness, but an advantage. And, when they are asked to report as though they have had it in the beginning, it not only asks them to deceive the reader, but also can limit the type of knowledge creation that qualitative research is best for (expert 11)

In a similar vein, one expert highlighted the critical, judgemental tones of retrospectively applied theory because of its connotations of self-disclosure:

I think that the labelling of level 4 needs to be reviewed as it’s very critical and it's unlikely that an author would select this level if you want to use it for authors to “self-identity” the use of theory in their research (expert 9)

Based on the expert panel's feedback we undertook an iterative process of revision to the typology. Our aim was to reflect on the thematic insights and adapt it in order to:

• Make the typology less hierarchical and value-laden;
• Capture the complex relationship between theory and qualitative research;
• Account for how both inductive and deductive approaches might be used;
• Allow for different types (including single or multiple theories) to be drawn upon within one study;
• Legitimise the utilisation of theory at different points of the research process.

The discussion section presents the new, QUANTUM typology that takes account of these essential revision points.

## 4 Discussion

Table 5 presents the QUANTUM typology that is grounded in the expert views as presented earlier. From a structural viewpoint, the most apparent change is an attempt to make it less hierarchical. Presented in landscape, the content sits side-by-side - as opposed to stacked as it was in portrait format - to imply complementarity, rather than hierarchy. We have also withdrawn reference to ‘levels’ which was deemed to be too judgemental and emphasised the values that were both implicit and explicit in the original. Again, in terms of structure, the typology is now divided into two sections. The three left-hand columns are concerned with the visibility of theory and prompt the reader of a qualitative report to question the extent to which they can see a theory, or indeed theories, in the work. As shown, this may fall under one of three options: seemingly absent, partially described or consistently described. Under the latter two, we have expanded on how the theory or theories might be evident, providing far more options than the original, acknowledging the multiplicity of ways that theory might be seen in qualitative research. We have removed ‘retrospectively applied’ because this was considered to be of limited practical use. Inspired by Green and Evans' (2004) notion of ‘post-hocery’, the revised typology acknowledges the use of theory at different stages of the research process and the level 4, retrospectively applied theory has been removed. Post-hoc application of theory now has a legitimate place. We have also collapsed ‘implied’ and ‘partially applied’ into the single and simpler, ‘partially described’. We have deliberately used the terms ‘Partially *described*’ and ‘Consistently *described*’ to mirror the language used in the right-hand column which is concerned with description of theory.

**Table 5:** The QUANTUM typology.

The visibility of theory			The description of theory		
Question: How well are you able to ‘see’ theory?			Question: How do authors describe their use of theory?		
Seemingly absent	Partially described	Consistently described	How theory has informed the study	Where theory is located within the study	How theory interacts with methodology
A.1. Theory is not mentioned at all.	B.1. Theory (or theories) may be mentioned or discussed with reference to theorists in the field, but no explicit statement is made about the influence of these on the study. B.2. It is not clear how theory and methodology are related.	C.1. The article is infused with theory. C.2. Theory is consistently and clearly described throughout the entire research process. C.3. Theory guides and directs the various phases of the research process and can be tracked throughout a published article. C.4. Theory is addressed in relation to the alignment of literature, research questions, methods, analysis and findings.	D.1. The study may be described as empirical (inductive) research. D.2. The authors may draw on a single theory. D.3. The authors may blend multiple theories. D.4. The appropriateness of the theory or theories is critiqued.	E.1. Theory may be evident from the beginning and guide the research questions. E.2. A theoretical lens may be identified during the study and is used as an analytical framework. E.3. A single theory or the work of multiple theorists may be utilised near the end of a study to make sense of the study findings. E.4: Theory (single or multiple) has been rigorously applied to all stages of the research.	F.1. Theory may be derived from the qualitative findings, as in a grounded theory study. F. 2. Researchers use their findings to further develop or critique existing theory.

As Thorne (2016) points out, the world of qualitative study is diverse and complex. When considering the relationship between theory and qualitative research, there are long-standing debates about how the two are connected (Keyton, Bisel, & Ozley, 2009). Through the expert views of our panel, it became clear, that a revised typology needed to reflect this complexity, capturing how inductive and deductive approaches need to be balanced, how theory might be used at different stages of a study, drawing perhaps of multiple theories.

Qualitative research holds that there is no observable reality and researchers utilising qualitative methods build findings inductively, from raw data to conceptual understanding (Garvey & Jones 2021). As MacFarlane and O'Reilly-de-Brún (2012) point out, the merits of highly inductive research designs in qualitative health research are well established. The potential tension though, is that operating within a particular theoretical perspective and using, for example, the constructs within it, often guides the analysis towards a more deductive, than inductive approach. The challenge is, how to balance the inherently inductive processes of qualitative analysis with the more deductive framework imposed by a theoretical framework. There are certainly critics of the latter. Tracy (2012) argues that deductive logic constrains theory, discourages grounded analyses and invites inappropriate benchmarks for quality. On the other hand, theoretical frameworks are purported to be useful in sensitising researchers to concepts they might not necessarily identify through inductive processes (MacFarlane & O'Reilly-de-Brún, 2012). Theoretical frameworks may be used to guide qualitative analysis by suggesting concepts and relationships to explore (Garvey & Jones 2021). ‘A researcher who cannot articulate a theoretical framework may not have done the difficult and essential work to unearth their deepest operating principles and preconceptions about their study’ (Collins & Stockton 2018, p. 2). We regard Sally Thorne's work on interpretive description as a helpful way of overcoming some of the potential tensions here. Interpretive description is an inductive analytical approach that was first described by Thorne, Reimer Kirkham and MacDonald-Emes (1997). It arose from a necessity to find a way to do applied qualitative research that could generate relevant and useful understandings of complex clinical phenomena. It thus has an emphasis on pragmatics. Importantly though, for informing debates about balancing inductive and deductive approaches, interpretive description is an approach to knowledge generation that straddles the chasm between objective neutrality and abject theorizing (Thorne 2016). Overall, we support the notion of working with theory and research (Jackson & Mazzei 2013; Meyer & Ward 2014). Working with theory is necessary because it teaches us that both data and theory have a supple substance. Data, theory and method never stand alone, but rather they ‘keep things on the move, keep things becoming’ (Jackson & Mazzei 2013, p. 270). This type of suppleness is the essence of what we have tried to embed within the QUANTUM typology.

In the original typology, we had argued for a gold standard in theory use as being level 5 (consistently applied). In other words, we held that the ‘best’ use of theory was when it was used at the beginning of a qualitative study to shape and inform all stages and processes. We still consider consistency to be an important marker of rigor. However, we have shifted our position as regards where and how theory might be used. We now join Meyer and Ward (2014) in being advocates of a pluralistic approach for theory verification and generation in qualitative research.

An important development and maturity in our thoughts since the publication of the original typology, is in the way that we conceptualise the analytical process. Reflecting our pluralistic viewpoint, we see theory and data as intertwined. Jackson and Mazzei (2013) describe how they use theory to link with their data and data to link with theory, which they refer to as ‘plugging in’. They ‘read the same data across multiple theorists by plugging the theory and the data into one another’ (Jackson & Mazzei 2013, p. 261). We are interested also, in their reference to using multiple theorists, which did not feature in the original typology, but is now legitimized within the QUANTUM typology.

A criticism of the original typology put forward by many of the experts was its limitations as regards allowing for theory development. As reported earlier, many referred to the specific case of grounded theory. In the context of a grounded theory study, Corbin and Strauss (2014) state a preference for conducting qualitative analysis without a theoretical framework, but they note that a theoretical framework may be useful in initial orientation. Moreover, reflecting on their own use of utilising a theoretical framework to guide open and axial coding in a grounded theory study, Garvey and Jones (2021) concluded that using theory in this way was useful and appropriate. With these things in mind, the QUANTUM typology makes a specific statement about the place of theory in grounded theory. We view this as an important addition to the typology.

To draw the discussion to a close, we return to the ADAPT-ITT framework that formed such a pivotal part in our project. The final two elements of the ADAPT-ITT framework are concerned with training and testing and to which we have not yet turned our attention. To begin this process, in July 2021 we undertook a follow-up exercise with the expert panel, asking for feedback and critique on the revised QUANTUM typology. All except one expert replied to the email inviting a response. From this, we made further revisions to the typology, developing it to the stage presented in this article. Going forward, we will welcome correspondence and feedback once the typology has been published. Over time, we will also have opportunity to elicit feedback from students who participate in our qualitative classes, in the same way as we have done for the original typology. We will thus be able to assess how the QUANTUM typology has been integrated and implemented at that stage and get a sense of its overall utility (or not) within the field of qualitative research.

### 4.1 Critical reflections

We are four researchers who have engaged variously - although differently – with qualitative research for what amounts to decades. We have adopted and adapted many theoretical perspectives in relation to our own qualitative work. We have an inherent bias towards theory in qualitative research and are readily drawn towards others who share this view. So, we support the contention that ‘to think with theory is not only useful, but *essential*, for without theory we have no way to think otherwise (Jackson & Mazzei 2013, p. 269). Likewise, we agree with Tracy's (2010) description of the importance of ‘rich rigor, whereby ‘a researcher with a head full of theories, and a case full of abundant data, is best prepared to see nuance and complexity’ (Tracy, p.841). But we are not blinkered to the limitations.

Silverman (2007) suggested that theory in qualitative research can lead to ‘ostentatious displays of theoretical virtuosity’ (p. 120). While this moral high ground may be a risk, there are some methodological limitations that we regard as far more important. Collins and Stockton (2018) highlight how over-reliance on theory may limit the ability to see emergent findings in the data. It may stifle inductive reasoning or ‘result in findings incongruent to the data’ (Garvey & Jones 2021, p. 1). We have previously referred to this as ‘squeezing the data'. Such challenges can of course be overcome. Garvey and Jones (2021) suggest that the risks in using a theoretical framework may be mitigated by exploring the fit between the data and the framework and ‘thoughtfully questioning when and how it will be used’ (p.5). They call upon researchers to adopt a stance of uncertainty and reflexivity; a viewpoint that is supported by others (Corbin & Strauss 2014), including ourselves.

As a final point of reflection, we turn to the work of Denzin (2017) who calls for critical qualitative research, where: ‘there is a need to unsettle traditional concepts of what counts as research, as evidence, as legitimate inquiry’ (p.8). As researchers from Europe, this has significance for all our qualitative research, but particularly that conducted in the Global South. Guzmán-Valenzuela and Barnett (2019) argue that debates about the relationship between theory and qualitative research have taken place mainly in Anglo-Saxon countries and has provided scant justice to an understanding of these issues in regions of the Global South. In their geopolitical analysis of published articles by Latin-American researchers, they conclude that theories in Latin America are mainly produced in the Global North. This leads them to call for new models of knowledge that are *from* and *for* the Global South (Guzmán-Valenzuela & Barnett 2019). Only one of the qualitative experts made explicit reference to this form of knowledge hegemony:

*An ongoing conversation … is examining the “whiteness” of certain strains of literature and concepts. Theory is certainly something that falls into this discussion as scholars often rely on White and Western ideas of what equates to theory. As such, I think it's important to consider non-white, non-western, or indigenous ideas when defining theory* (expert 11)

According to hooks (1994), theory has the capability of challenging the status quo and in the context of critical qualitative inquiry, it is crucial that theories respect ways of knowing that may be subjugated and marginalized. With this in mind point D.4. of the QUANTUM typology is concerned with the appropriateness of the theory or theories being used. We have added this as a prompt to qualitative researchers to reflect on the risks of perpetuating Western and Global North ways of knowing as privileged, leading to the marginalization and silencing of Global South and Indigenous knowledge systems.

## 5 Conclusion

We used the ADAPT-ITT framework as the structure for the revision to the original typology. The stepwise processes of ADAPT-ITT provided a framework through which we were able to critically reflect on the need to revise the typology in the first place, through the mechanisms by which the revisions were made, to the final testing and training required to evaluate it. In this article we presented the principal outcome of the process - the QUANTUM typology. Like its predecessor, we hope that it helps those who are designing qualitative studies to consider how and when theory might be used within their research. We expect that it will be a useful reference for writing or reading qualitative reports to critically evaluate the visibility and description of theory. Not so that it becomes yet another check-list, but so that it can be a reflexive aide in conducting and reporting qualitative research.

## Ethical statement

Ethical approval for this study was granted by the Science, Technology, Engineering and Mathematics Ethical Review Committee at the University of Birmingham.

## Declaration of competing interest

The authors declare no conflict of interest in relation to this manuscript.
